# Tooth brushing versus routine oral care impact on ventilator-associated pneumonia in PICU: a clinical trial

**DOI:** 10.1186/s12887-026-07239-x

**Published:** 2026-07-24

**Authors:** Ahmed R. Rezk, Samar A. Hassan, Rabab M. Abd Elhakam, Aya Mahmoud Kamel, Nehad A. Bakry

**Affiliations:** 1 Department of Pediatrics, Faculty of Medicine, Ain Shams University, Cairo, Egypt; 2 Orthodontics and Paediatric Dentistry department, Oral and Dental Research Institute, National Research Centre, Cairo, Egypt

**Keywords:** Pediatric intensive care unit (PICU), Ventilator-associated pneumonia (VAP), Oral care, Toothbrushing, Chlorhexidine

## Abstract

**Objectives:**

Ventilator-associated pneumonia (VAP) is a frequent and serious infection among mechanically ventilated children in pediatric intensive care units (PICUs), with oral colonization playing a key role in its pathogenesis. Effective oral hygiene measures are therefore crucial to reduce VAP incidence and improve clinical outcomes. This study aimed to compare the incidence of VAP in PICU patients receiving routine oral care versus toothbrushing with chlorhexidine, and to evaluate the impact on mechanical ventilation duration, PICU stay, and mortality with secondary outcomes including microbiological colonization, inflammatory markers, and inotropic support requirements.

**Design:**

A parallel-group superiority randomized controlled trial.

**Methods:**

A randomized controlled trial was conducted on 118 children aged 18 months to 16 years, including PICU patients requiring invasive mechanical ventilation for ≥ 48 h. On admission, patients were randomly allocated into two groups: the intervention group received oral care with toothbrushing plus 0.12% chlorhexidine mouth rinse, while the control group received routine care with 0.12% chlorhexidine rinse only. Both procedures were performed three times daily. VAP was diagnosed according to CDC criteria. Quantitative and qualitative analyses assessed variables distinguishing VAP cases from controls, and multivariate logistic regression identified independent predictors of disease severity.

**Results:**

The results of our study revealed that VAP occurred in 31 patients (52.5%) in the control group and 25 patients (42.3%) in the intervention group, indicating a lower incidence with toothbrushing plus chlorhexidine, although this difference was not statistically significant (*p* = 0.36). Among all participants, 45.8% died and 54.2% were discharged. Mortality was higher in the control group (53.7%) compared with the intervention group (46.3%), while discharge rates favored the intervention group (53.1% vs. 46.9%); however, these differences did not reach statistical significance (*p* = 0.46). Positive sputum cultures were more frequent in the control group (55.6%) than in the intervention group (44.4%), whereas negative cultures were more common among intervention patients (54.7% vs. 45.3%), with no significant difference between groups (*p* = 0.268).

**Conclusion:**

Although adding toothbrushing to routine chlorhexidine oral care did not produce a statistically significant reduction in VAP incidence or mortality, a consistent trend toward clinical benefit was observed across multiple outcome measures, including lower rates of positive sputum cultures, reduced inotropic support requirements, and fewer new pulmonary infiltrates. These findings highlight the potential value of enhanced oral hygiene in the PICU and support the need for larger multicenter studies to confirm its effect.

**Trial registration:**

ClinicalTrials.gov, NCT07287566. Registered on November 20, 2025. Retrospectively registered at https://clinicaltrials.gov/ct2/show/NCT07287566 .

**Protocol and statistical analysis plan access:**

The full study protocol and the statistical analysis plan are included in this document and are available from the corresponding author upon reasonable request.

**Supplementary Information:**

The online version contains supplementary material available at 10.1186/s12887-026-07239-x.

## Introduction

Ventilator-associated pneumonia (VAP) is a significant complication in critically ill children. According to the Centers for Disease Control and Prevention (CDC) and the National Healthcare Safety Network (NHSN), it is defined as new, persistent radiographic infiltrates with worsening gas exchange in children who have been mechanically ventilated for at least 48 h, accompanied by at least three of the following clinical criteria: unexplained temperature instability, leukopenia, changes in the character of respiratory secretions, respiratory distress, and bradycardia or tachycardia [1].

VAP is the second most common hospital-acquired infection in PICUs, representing 7–32% of healthcare-associated infections and 10% of pediatric device-related infections [2]. Its development is associated with prolonged mechanical ventilation, longer PICU stays, higher healthcare costs, and increased morbidity and mortality.

Oral colonization is a key factor in VAP pathogenesis, making effective oral hygiene essential. Structured oral care protocols aim to reduce infectious and inflammatory complications through mechanical biofilm removal by tooth brushing and pharmacologic antiseptics such as chlorhexidine [3]. Despite multiple studies on oral hygiene interventions, evidence in children remains inconsistent, particularly regarding combined regimens of tooth brushing and antiseptic use. This gap underscores the need for further investigation.

This study evaluates the impact of different oral hygiene methods on the incidence of VAP in mechanically ventilated children. It compares standard care using 0.12% chlorhexidine alone with an enhanced regimen combining tooth brushing and chlorhexidine. Secondary outcomes include duration of mechanical ventilation, length of PICU stay, mortality, and laboratory parameters such as C-reactive protein (CRP), total leukocyte count (TLC), and the need for inotropic support.

## Patients and materials

This study was designed as a parallel-group superiority randomized controlled trial conducted in the PICU at Ain Shams University Children’s Hospital between June 2024 and July 2025 and was reported in accordance with the Consolidated Standards of Reporting Trials (CONSORT) guidelines. A total of 118 mechanically ventilated children who fulfilled the inclusion criteria were enrolled and randomly assigned to either an intervention or a control group. All patients received standard PICU care, including the ventilator-associated pneumonia prevention bundle, in addition to the assigned oral care intervention. The intervention group received oral hygiene with toothbrushing combined with 0.12% chlorhexidine mouth rinse. Patients were positioned in a semi-recumbent posture with the head of the bed elevated 30°–45°. Oral care was performed three times daily while on invasive mechanical ventilation. Each session lasted at least five minutes and consisted of gentle brushing using a soft toothbrush and toothpaste, employing the Bass technique (the brush positioned at a 45° angle with firm but gentle pressure and short back-and-forth or circular motions, repeated 15–20 times per area for approximately one minute). This was followed by application of 0.12% chlorhexidine solution using a swab to the oral mucosa, teeth, gums, hard palate, and tongue, then rinsed with 20 mL of normal saline. The procedure concluded with hypopharyngeal suctioning. The control group received routine oral care with 0.12% chlorhexidine mouth rinse alone, applied using a swab to the tongue, gums, and mucosa, followed by 20 mL of normal saline and hypopharyngeal suctioning. Both oral care regimens were performed three times daily. VAP diagnosis was based on the CDC/NHSN pediatric clinical definition, requiring new persistent radiographic infiltrates with worsening gas exchange in patients mechanically ventilated for ≥ 48 h, accompanied by at least three of the following criteria: temperature instability with no other recognized cause, leukopenia, change in the character of respiratory secretions, respiratory distress, and bradycardia or tachycardia. It should be noted that this definition predates the current CDC Ventilator-Associated Events (VAE) surveillance framework, which classifies events along a spectrum (VAC → IVAC → Possible/Probable VAP). Retrospective reclassification was not feasible, as it would require re-abstraction of ventilator settings data not prospectively collected [14]. Inclusion criteria comprised children aged 18 months to 16 years with respiratory distress requiring invasive mechanical ventilation (MV) via endotracheal intubation. Exclusion criteria included immune deficiency, coagulation disorders, and jaw abnormalities or fractures. Detailed medical history was obtained from guardians and patient records at admission. Demographic data, vital signs, and chest examination findings were recorded at baseline and on the fifth day of ventilation. Laboratory investigations included complete blood count with differential, CRP, venous blood gases (VBG). Sputum samples were obtained on the fifth day via blind broncho-alveolar lavage (BAL). Chest radiographs were performed on admission and repeated on the fifth day to identify new infiltrates or consolidations suggestive of VAP.

Adverse events related to oral care procedures were actively monitored clinically throughout the study period, and none were observed or reported.

Patients and the public were not involved in the design, conduct, reporting, or dissemination plans of this study.

### Sample size calculation

The sample size was calculated using G*Power software based on an anticipated difference in proportions between the two groups. A medium effect size was assumed due to limited pediatric data. With a two-sided alpha level of 0.05 and a power of 80%, the required sample size was estimated to be 59 participants per group.

### Training and fidelity

Prior to study initiation, a pediatric dentist trained the PICU nursing staff on the standardized oral care protocol. The correct brushing technique, intraoral massaging, and suctioning techniques, as well as the use of saline and chlorhexidine rinses were all included in the training. To ensure consistency, adherence was monitored throughout the trial.

### Randomization

Random assignment (1:1 ratio) was used to place participants in either the intervention or control groups.

Participants were enrolled by PICU physicians, and group allocation was performed using sealed envelopes by an independent investigator. The personnel enrolling participants did not have access to the random allocation sequence.

### Blinding

Because of the nature of the intervention, it was not possible to blind the nursing staff, as they were aware of whether toothbrushing or swabbing was performed. The patients were mechanically ventilated and therefore unaware of group allocation. Outcome assessment and statistical analysis were carried out by individuals who were blinded to the intervention groups. The study was conducted as a single blind randomized controlled trial with blinded outcome assessment.

### Sample collection and laboratory analysis

Serum and BAL samples were collected on the fifth day following initiation of invasive mechanical ventilation.

### Blood sample collection

Two milliliters of venous blood were drawn and transferred into plain tubes. The samples were allowed to clot for 10–20 min at room temperature, then centrifuged at 2,000–3,000 rpm for 20 min. The resulting serum supernatant was carefully separated from the cellular sediment and stored for analysis. Routine investigations including complete blood count (CBC) with differential, CRP, and VBG were performed for all patients.

### BAL sample collection

A catheter was advanced through the endotracheal tube beyond the carina; saline was instilled and aspirated gently to collect the lavage fluid for microbiological culture. This method was selected for its clinical feasibility and suitability for use in mechanically ventilated pediatric patients without requiring specialized bronchoscopy equipment or additional sedation. However, blind BAL carries recognized limitations: it does not allow direct visual confirmation of the sampling site, may yield samples from non-dependent lung segments, and carries a higher risk of upper airway contamination compared to bronchoscopic BAL. These factors may have influenced the specificity of microbiological findings and should be considered when interpreting culture results. Microbiological evaluation included identification of microorganisms isolated from blind broncho-alveolar lavage (BAL) cultures, along with their antibiotic susceptibility profiles. BAL specimens were directly inoculated into selective and non-selective culture media. Incubation: MacConkey agar plates were incubated at 37 °C under aerobic conditions, and blood agar plates were incubated at 37 °C with 5% CO₂. Identification: Direct smears were stained with Gram stain for preliminary evaluation and treatment guidance. Additional smears were prepared from bacterial growth for confirmatory staining. Antimicrobial susceptibility testing: Microorganisms were identified by standard spot and biochemical tests. Susceptibility testing was performed using the Kirby–Bauer disc diffusion method on Mueller–Hinton agar plates, with bacterial suspensions adjusted to a 0.5 McFarland turbidity standard.

### Outcomes

The primary outcome was the incidence of ventilator-associated pneumonia (VAP). Day 5 of mechanical ventilation was selected as the primary outcome evaluation timepoint based on the epidemiological classification of VAP. Early-onset VAP occurs within the first 4–7 days of ventilation and represents the period of greatest colonization risk. This timepoint provides a standardized assessment window capturing early-onset events while allowing sufficient time for VAP to manifest following the 48-hour definitional threshold. Clinical deterioration, worsening radiographic findings, or new culture positivity arising at any other point during the PICU stay were also recorded and contributed to the broader secondary outcomes analysis. Primary outcome data were available for all randomized participants in both groups (59/59 in each group).

Secondary outcomes included duration of mechanical ventilation, length of PICU stay, and mortality.

### Statistical analysis

All data were coded, organized, and analyzed statistically using IBM SPSS Statistics version 29.0 (IBM Corp., Armonk, NY, USA). The Shapiro–Wilk test was applied to assess the normality of quantitative variables. Normally distributed data are presented as mean ± standard deviation (SD), while non-normally distributed data are expressed as median with interquartile range (IQR). Categorical variables are reported as frequencies and percentages. Comparisons between the two study groups were conducted using the independent sample t-test for parametric quantitative data and the Mann–Whitney U test for non-parametric data. The chi-square test (χ²) or Fisher’s exact test was used to compare categorical variables, as appropriate. Associations between continuous variables were evaluated using Pearson’s or Spearman’s correlation coefficients, based on data distribution. A p-value of less than 0.05 was considered statistically significant. All randomized participants were included in the analysis according to the intention-to-treat principle.

Missing data was handled using complete case analysis, and no missing data were identified for the primary outcome. Ventilator-free days at day 28 (VFD-28) and PICU-free days at day 28 (PFD-28) were retrospectively calculated as post-hoc analyses to account for competing risks from early mortality. VFD-28 was defined as 28 minus the number of days on mechanical ventilation for survivors, and zero for patients who died. PFD-28 was defined analogously using PICU length of stay. These metrics were compared between groups using the Mann–Whitney U test.

A data monitoring committee was not established due to the low-risk nature of the intervention.

### Ethical considerations

The study was approved by the Faculty of Medicine, Ain Shams University Research Ethics Committee (FMASU REC No. FMASU MS 407/2024; FWA No. FWA000017585) (Appendix 1).

Written informed consent was obtained from the parents or legal guardians of all participants in accordance with the Declaration of Helsinki (2013 revision) (Appendix 2).

## Results

In the period from June 2024 to July 2025, 118 patients were included in this study and randomized to control group (routine oral care group) or intervention group (tooth brushing with chlorhexidine group). There were 59 patients in each group. Table 1 shows the clinical characteristics of the patients at admission.

**Table 1:** Baseline demographic and clinical characteristics of PICU patients at admission in the control and intervention groups

Characters	Control group (*n* = 59) Mean ± SD	Intervention group (*n* = 59) Mean ± SD
Sex		
Male	27 (45.8%)	32 (54.2%)
Female	32 (54.2%)	27 (45.8%)
Age (years)	5.22 ± 4.44	5.89 ± 4.73
Admission diagnosis		
Surgical patient	9 (60.0%)	6 (40.0%)
Cardiovascular disease with heart failure	4 (40.0%)	6 (60.0%)
Respiratory disease		
➢ Pneumonia	22 (51.2%)	21 (48.8%)
➢ Bronchiolitis	1 (33%)	2 (66.7%)
➢ Lung mass	1 (33%)	1 (50%)
Renal disease		
➢ Acute kidney injury on top of chronic kidney disease	5 (83.3%)	1 (16.7%)
➢ Nephrotic syndrome	2 (66.7%)	1 (33.3%)
➢ Lupus nephritis	0 (0.0%)	1 (100.0%)
➢ Renal failure	0 (0.0%)	2 (100.0%)
Sepsis	6 (60.0%)	4 (40.0%)
Disturbed conscious level	2 (33.3%)	4 (66.6%)
Secondary Hemophagocytic lympho-histiocytosis	1 (25.0%)	3 (75.0%)
Inborn error of metabolism	1 (50.0%)	1 (50.0%)
Diabetic ketoacidosis (DKA)	2 (66.7%)	1 (33.3%)
Hepatic Encephalopathy	1 (25.0%)	3 (75.0%)
Neurological disease	2 (66.7%)	1 (33.3%)
Auto-immune hemolytic anemia	0 (0.0%)	1 (100.0%)
Comorbidity		
No	44 (51.1%)	43 (48.8%)
Cardiovascular disease with heart failure	2 (66.7%)	1 (33.3%)
Renal disease	4 (80.0%)	1 (20.0%)
Leukemia	5 (50.0%)	5 (50.0%)
Secondary Hemophagocytic lympho-histiocytosis	1 (50.0%)	1 (50.0%)
Inborn error of metabolism	0 (0.0%)	1 (100.0%)
Neurological disease	1 (50.0%)	1 (50.0%)
Down syndrome	2 (40.0%)	3 (60.0%)
Cerebral palsy (CP)	0 (0.0%)	2 (100.0%)
Anemia	0 (0.0%)	1 (100.0%)

n: Number, Percentages may not total 100% due to rounding

**Fig. 1:**
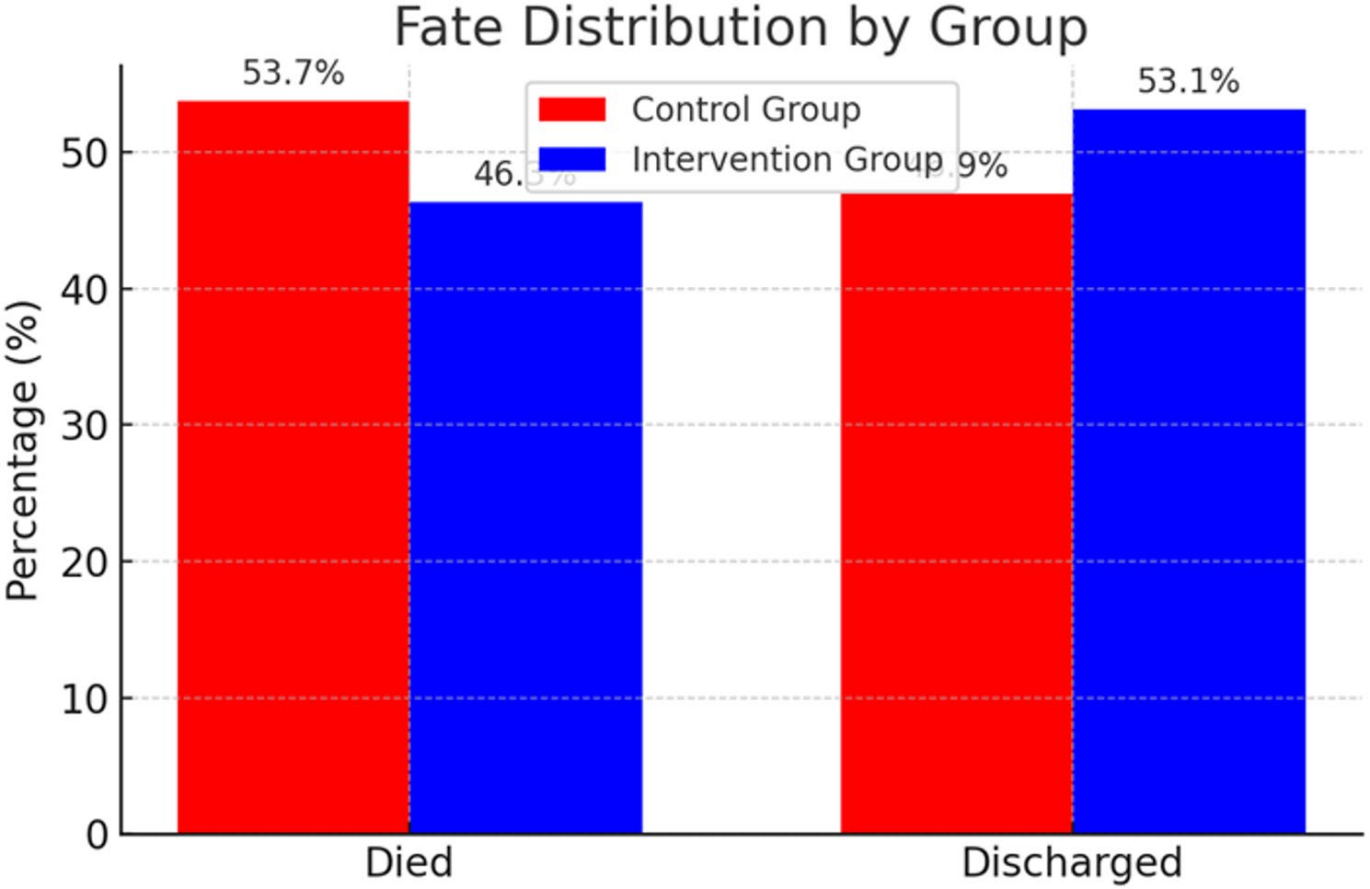
Clinical outcome distribution in study population comparing the control and intervention groups. The control group showed a mortality rate of 53.7% and a discharge rate of 46.3%, whereas the intervention group showed a mortality rate of 46.9% and a discharge rate of 53.1%

The difference in the duration of mechanical ventilation was not statistically significant (*p* = 0.229). The mean duration of admission was slightly longer in the intervention group (13.15 ± 10.33 days) than in the control group (12.00 ± 8.42 days), but this difference was not statistically significant (*p* = 0.793). Among the total 118 participants, 54 (45.8%) died and 64 (54.2%) were discharged. In the control group, 53.7% of patients died, whereas 46.3% died in the intervention group. Conversely, a higher percentage of discharges was observed in the intervention group (53.1%) than in the control group (46.9%) (Fig. 1). Despite this trend suggesting a modest survival advantage in the intervention arm, the difference was not statistically significant, as indicated by a p-value of 0.46 (Table 2).

**Table 2:** Comparison of mechanical ventilation duration, PICU length of stay, and clinical outcomes between the control and intervention groups

Events	Control group (*n* = 59)	Intervention group (*n* = 59)	*P*-value *
Duration of mechanical ventilation (Mean ± SD)	5.53 ± 2.16	6.12 ± 2.41	0.229
Length of PICU stay (Mean ± SD)	12.00 ± 8.42	13.15 ± 10.33	0.793
Fate			
Discharge	30 (46.9%)	34 (53.1%)	0.46
Death	29 (53.7%)	25 (46.3%)	
Ventilator-free days at day 28 (VFD-28) Mean ± SD	11.97 ± 11.92	12.95 ± 11.34	0.959
PICU-free days at day 28 (PFD-28) Mean ± SD	9.56 ± 10.07	8.17 ± 9.37	0.430

* P-value ≤0.05 was considered statistically significant (95% confidence interval)

❖ Percentages may not total 100% due to rounding

Among the total 118 patients, the majority (85.6%) tested positive for CRP, indicating widespread systemic inflammation across the cohort. The proportion of CRP-positive patients was nearly identical between the groups (50.5%) in the control group and (49.5%) in the intervention group. Similarly, negative CRP results were slightly more common in the intervention arm (52.9%) than in the control arm (47.1%), but the overall number of negative cases remained low (14.4%). The p-value was 0.793, confirming that there was no statistically significant difference in CRP status between the two groups. These findings suggest a balanced inflammatory profile at baseline.

**Fig. 2:**
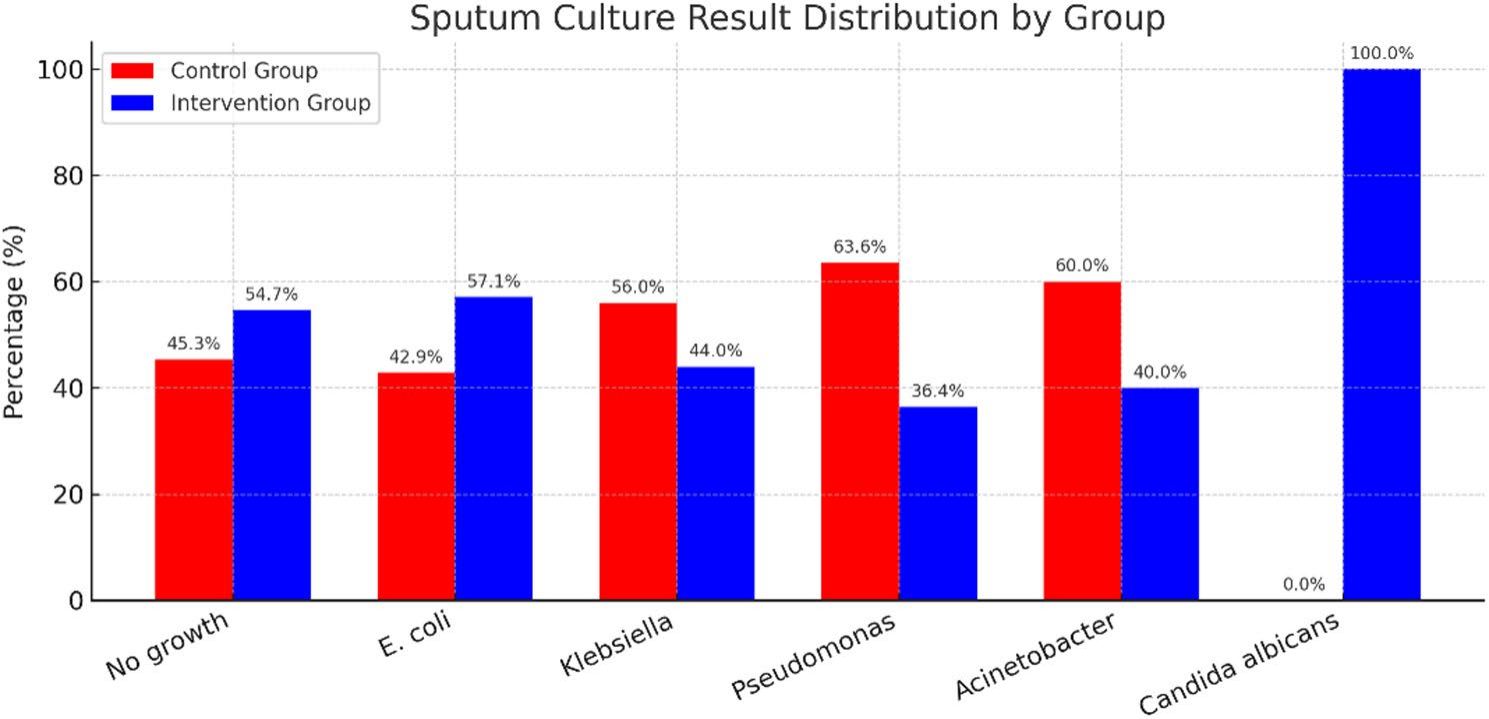
Distribution of sputum culture results among the study population comparing the control and intervention groups

With respect to the development of new pulmonary infiltrations, 44.1% of patients were affected, with a higher incidence in the control group (55.8%). Among those without new infiltrations (55.9%), tooth brushing with chlorhexidine was more common (54.5%). Most samples (54.2%) had no microbial growth, with the intervention group having a slightly higher percentage (54.7%) than the control group (45.3%). Although a variety of pathogens were present in both groups, the control group had slightly greater bacterial growth, especially the multidrug resistant group (Fig. 2). Inotropic support was necessary for 38.1% of all patients, with a higher percentage in the control group (57.8%) compared to the intervention group (42.2%). This suggests a greater need for cardiovascular support in the control arm. The mean number of days with worsening respiratory symptoms while on MV was greater in the control group, although it did not differ significantly between the groups (*p* = 0.232). These results suggest that the intervention group may have experienced a lower burden of clinical deterioration related to hemodynamic instability and respiratory complications (Table 3).

**Table 3:** Comparison of inflammatory markers, radiological findings, microbiological results, hemodynamic support, and respiratory deterioration between the control and intervention groups

Events	Control group (*n* = 59)	Intervention group (*n* = 59)	*P*-value *
CRP			
Negative	8 (47.1%)	9 (52.9%)	0.793
Positive	51 (50.5%)	50 (49.5%)	
New x-ray infiltrates			
No	30 (45.5%)	36 (54.5%)	0.26
Yes	29 (55.8%)	23 (44.2%)	
Sputum culture			
➢ No growth	29 (45.3%)	35 (54.7%)	0.268
➢ Growth	30 (55.6%)	24 (44.4%)	
• *E. coli*	3 (42.9%)	4 (57.1%)	
• *Klebsiella*	14 (56.0%)	11 (44.0%)	
• *Pseudomonas*	7 (63.6%)	4 (36.4%)	
• *Acinetobacter*	6 (60.0%)	4 (40.0%)	
• Candida albicans	0 (0.0%)	1 (100.0%)	
Inotropic support			
No	33 (45.2%)	40 (54.8%)	0.18
Yes	26 (57.8%)	19 (42.2%)	
Days of worsening of respiratory symptoms Mean ± SD	1.88 ± 1.97	1.44 ± 1.99	0.232

*E. coli* Escherichia Coli

* P-value ≤ 0.05 was considered statistically significant (95% confidence interval)

❖ Percentages may not total 100% due to rounding

Among all participants, mortality was significantly greater in those with positive sputum cultures (61.1%) than in those with no growth (32.8%), with a p-value of 0.002, indicating a statistically significant association between positive culture results and death. When analyzed by group, this association remained significant in the control group. Patients with culture growth in the control arm presented a notably higher mortality rate (66.7%) than did those without growth (31.0%), with a p-value of 0.006. However, in the intervention group, while a similar trend was observed in mortality among those with culture-positive results (54.2% vs. 34.3%), the difference was not statistically significant (*p* = 0.129). These results suggest that a positive sputum culture could indicate a poorer prognosis, especially in the control group (Table 4).

**Table 4:** Association between sputum culture results and mortality in the overall cohort and stratified by study group

Sputum culture result	Died *n* (%)	Discharged *n* (%)	*P*-value *
No growth	21 (32.8%)	64 (54.2%)	0.002
Growth	33 (61.1%)	21 (38.9%)	
Control group			
No growth	9 (31.0%)	20 (69.0%)	0.006
Growth	13 (54.2%)	11 (45.8%)	
Intervention group			
No growth	12 (34.3%)	23 (65.7%)	0.129
Growth	13(54.2%)	11 (45.8%)	

* P-value ≤0.05 was considered statistically significant (95% confidence interval)

❖ Percentages may not total 100% due to rounding

In the control group, mortality rates were nearly identical between males (48.1%) and females (50.0%), with a p-value of 0.887, indicating no statistically significant difference. In the intervention group, however, a notable difference emerged: male patients had a considerably lower mortality rate (31.3%) compared to females (55.6%). Although this trend suggests a potential gender-related survival advantage for males in the intervention group, the association did not reach statistical significance (*p* = 0.06). Overall, while gender did not significantly affect outcomes in either group, the near-significant result in the intervention arm may warrant further investigation in a larger cohort (Table 5).

**Table 5:** Sex-based differences in mortality and discharge outcomes within each study group

Group	Sex	Died *n* (%)	Discharged *n* (%)	Total *n* (%)	Chi²	*p*-value	significant
Control Group	Male	13 (48.1%)	14 (51.9%)	27 (45.8%)	0.02	0.887	N.S
	Female	16 (50.0%)	16 (50.0%)	32 (54.2%)			
	Total	29 (49.2%)	30 (50.8%)	59 (100.0%)			
Intervention Group	Male	10 (31.3%)	22 (68.8%)	32 (54.2%)	3.54	0.06	N.S
	Female	15 (55.6%)	12 (44.4%)	27 (45.8%)			
	Total	25 (42.4%)	34 (57.6%)	59 (100.0%)			
Statistical test used: Chi-square test							
p-value ≤ 0.05 considered statistically significant (95% confidence interval).							

*N* number, *% *percentage, *Chi²* Chi-square, *CI* Confidence Interval, *p-value* Probability value, *NS* not Significant

* P-value ≤0.05 was considered statistically significant (95% confidence interval)

❖ Percentages may not total 100% due to rounding

Patients with positive sputum cultures demonstrated significantly worse outcomes across all clinical parameters compared to culture-negative patients (Table 6). The duration of mechanical ventilation was significantly longer in culture-positive patients (6.78 ± 2.16 days vs. 4.98 ± 2.10 days; *p* < 0.001), as was PICU length of stay (15.38 ± 10.29 days vs. 11.21 ± 7.77 days; *p* = 0.023). Inotropic support was required significantly more often in culture-positive patients (52.7% vs. 25.4%; *p* = 0.004). New pulmonary infiltrates on follow-up chest radiograph were markedly more prevalent in the growth group (89.1% vs. 3.2%; *p* < 0.001). CRP positivity was also significantly higher in culture-positive patients (96.4% vs. 77.4%; *p* = 0.007). Mortality was significantly greater in culture-positive patients (61.8% vs. 31.7%; *p* = 0.002), consistent with the previously reported association between microbiological infection burden and adverse PICU outcomes.

**Table 6:** Clinical outcomes stratified by sputum culture result in the overall study cohort

Variable	No Growth (*n* = 63)	Growth (*n* = 55)	*P*-value *
Duration of MV (days) — Mean ± SD	4.98 ± 2.10	6.78 ± 2.16	< 0.001*
PICU length of stay (days) — Mean ± SD	11.21 ± 7.77	15.38 ± 10.29	0.023*
Inotropic support — n (%)	16 (25.4%)	29 (52.7%)	0.004*
New pulmonary infiltrates on CXR — n (%)	2 (3.2%)	49 (89.1%)	< 0.001*
CRP positive — n (%)	48 (77.4%)	53 (96.4%)	0.007*
Mortality — n (%)	20 (31.7%)	34 (61.8%)	0.002*

*MV* Mechanical ventilation, *PICU* Pediatric intensive care unit, *CXR* Chest X-ray, *CRP* C-reactive protein

* P-value ≤ 0.05 considered statistically significant. Mann–Whitney U test used for continuous variables, Chi-square test for categorical variables. bold values indicate statistically significant results.

Univariate logistic regression identified three significant predictors of mortality: positive sputum culture (OR = 3.48; 95% CI: 1.63–7.44; *p* = 0.001), inotropic support (OR = 15.24; 95% CI: 5.97–38.90; *p* < 0.001), and new pulmonary infiltrates (OR = 3.44; 95% CI: 1.61–7.39; *p* = 0.001). Sex, age, and group allocation were not significant on univariate analysis (Table 7). On multivariate analysis adjusting for all three significant variables, inotropic support emerged as the only independent predictor of mortality (aOR = 13.02; 95% CI: 5.00–33.93; *p* < 0.001). Positive sputum culture and new pulmonary infiltrates did not retain independent significance after adjustment, suggesting that their effect on mortality is mediated through hemodynamic instability. Sex was not a significant predictor on either univariate (*p* = 0.141) or multivariate analysis.

**Table 7:** Univariate and multivariate logistic regression analysis for predictors of mortality

Variable	Univariate Analysis			Multivariate Analysis		
	OR	95% CI	*P*-value	aOR	95% CI	*P*-value
Sex (Female)	1.73	0.83–3.60	0.141	—	—	—
Age (years)	0.99	0.92–1.08	0.876	—	—	—
Group (Intervention)	0.76	0.37–1.57	0.460	—	—	—
Positive sputum culture	3.48	1.63–7.44	0.001*	2.14	0.39–11.82	0.384
Inotropic support	15.24	5.97–38.90	< 0.001*	13.02	5.00–33.93	< 0.001*
New pulmonary infiltrates	3.44	1.61–7.39	0.001*	1.15	0.20–6.46	0.875

*OR* Odds ratio, *aOR* Adjusted odds ratio, *CI* Confidence interval

* P-value ≤ 0.05 considered statistically significant. Multivariate model includes variables with *p* < 0.1 on univariate analysis (positive sputum culture, inotropic support, new pulmonary infiltrates). — indicates variable not entered in the multivariate model. bold values indicate statistically significant results.

## Discussion

Ventilator-associated pneumonia (VAP) remains one of the most serious complications in PICUs, contributing to increased morbidity, mortality, and healthcare costs among critically ill children [4].

Given that the oropharynx serves as a primary reservoir for pathogenic organisms that can be aspirated into the lower respiratory tract, the importance of oral hygiene in VAP prevention has been increasingly emphasized [5]. The present study aimed to evaluate the impact of different oral hygiene methods specifically routine chlorhexidine swabbing versus tooth brushing combined with chlorhexidine mouthwash on the incidence and outcomes of VAP in pediatric patients admitted to the PICU. The analysis encompassed a variety of demographic, clinical, microbiological, and outcome parameters across 118 children randomized into control and intervention groups.

The demographic data confirmed successful randomization. Both groups had nearly identical mean ages (5.22 years in the control group vs. 5.89 in the intervention group) and an even gender distribution (50% each). Notably, the CRP positivity rate, a marker of systemic inflammation, was similarly high in both arms (85.6%), further supporting baseline equivalence.

The demographic balance and baseline comparability observed in our study are consistent with findings from prior research. In the study by Vidal et al. [6], patients were similarly well-matched at baseline in terms of age, gender, and clinical parameters, ensuring that differences in outcomes could be attributed to the oral hygiene intervention rather than pre-existing disparities.

Although there was a wide range of initial diagnoses, respiratory diseases were the most prevalent (40.7%), with pneumonia accounting for most respiratory presentations (89.6%). Although the control group had a slightly higher burden of surgical and renal diagnoses, the distributions of other conditions, such as sepsis, cardiovascular disease, and renal disease, were balanced. Crucially, despite minor differences in certain conditions, the prevalence of comorbidities such as leukemia, Down syndrome, and cerebral palsy was low and did not exhibit any statistically significant clustering.

The diversity of initial diagnoses observed in the current study, with respiratory diseases especially pneumonia, being the most prevalent, is consistent with findings from several supporting studies. According to Bhattacharya et al. [2], pneumonia and other respiratory tract infections are the most common primary diagnoses among pediatric intensive care unit patients and are a major cause of the development of VAP.

Additionally, Gomaa et al. [7] reported that a significant percentage of the critically ill pediatric population had respiratory illnesses, particularly pneumonia, during their evaluation of an oral hygiene protocol in an Egyptian PICU. The distribution of primary diagnoses and related comorbidities in the current study is consistent with these studies, which collectively support the prevalence of respiratory conditions in children on mechanical ventilation.

The VAP incidence observed in this study (52.5% in the control group and 42.3% in the intervention group) may appear elevated; however, these rates are consistent with data from comparable high-burden PICU settings. Bhattacharya et al. [2] reported VAP rates of 36–52% in developing-country pediatric ICUs, and Gomaa et al. [7] reported rates exceeding 50% in an Egyptian PICU, reflecting the combined burden of critical illness severity, limited infection control resources, and predominantly respiratory diagnoses. Although both were marginally higher in the intervention group, there were no appreciable differences in the total length of hospital stay (13.15 vs. 12.00 days) or the number of days on mechanical ventilation (6.12 days in the intervention group vs. 5.53 days in the control group). Compared with the control group (46.9% discharge, 53.7% mortality), the intervention group had a higher discharge rate (53.1%) and a lower mortality rate (46.3%). This difference suggests a potential clinical benefit of the intervention, even though it was not statistically significant (*p* = 0.46).

Although the difference in mortality was not statistically significant, Kusahara et al. [8] reported that patients in the chlorhexidine group, especially those without early oropharyngeal colonization, had fewer episodes of VAP, which is strongly associated with improved survival outcomes. This was observed in a double-blind randomized trial of critically ill children. Despite a trend toward decreased pathogen colonization in the intervention group, there was no discernible difference in the length of hospital or intensive care unit stay between the chlorhexidine and placebo groups. Also, Silva et al. [9] emphasized that combining tooth brushing with chlorhexidine as opposed to using chlorhexidine alone was associated with reduced VAP incidence and showed favorable trends in mortality and ICU outcomes across multiple trials. These findings underscore the connection between improved oral care, reduced microbial burden, and better clinical outcomes, reinforcing the significance of our study’s finding that infection, as evidenced by positive sputum cultures, was significantly correlated with mortality (*p* = 0.002).

CRP levels were similarly elevated across both groups, highlighting the systemic inflammatory response associated with critical illness. The control group had higher rates of inotropic support and new pulmonary infiltrates on chest X-ray, two alternative indicators of disease severity. In particular, 55.8% of the control patients experienced new infiltrates, compared with 44.2% in the intervention group, and 57.8% of the control patients needed inotropes, compared to 42.2% in the intervention group. These findings indicate that improved oral hygiene could help lower the chance of systemic deterioration or the development of severe VAP. According to the sputum culture results, the intervention group had a greater proportion of negative cultures (54.7%) than did the control group (45.3%). The most prevalent pathogen among positive cultures was Klebsiella, followed by Pseudomonas and Acinetobacter. The control group experienced a higher incidence of culture-positive VAP, which is consistent with the literature indicating that mechanical debridement through tooth brushing improves oral hygiene and may lessen microbial colonization. Although the trend supported the intervention, the difference in positive culture rates between groups did not reach statistical significance (*p* = 0.268). Curiously, the intervention group experienced an earlier mean number of days with worsening respiratory symptoms while on MV (1.44 vs. 1.88), which might indicate better detection or earlier onset of complications rather than actual deterioration.

According to Pinto et al. [10], the impact of oral hygiene interventions is more evident in localized infection control and outcomes such as VAP incidence and mortality, even though they may not directly affect systemic inflammatory markers such as CRP.

Additionally, Vidal et al. [6], reported that patients who received tooth brushing combined with chlorhexidine had a significantly lower incidence of VAP than did those who received chlorhexidine alone. This reduced infection burden likely contributes to fewer complications, such as septic shock or systemic inflammation, which are common triggers for inotropic support and radiographic lung changes.

Additionally, Zhao et al. [11] in a Cochrane systematic review found that tooth brushing combined with antiseptics likely reduces the incidence of VAP compared to antiseptics alone, pointing to the crucial role of biofilm disruption.

Similar to the microbiological profile observed in our study, Kusahara et al. [8] demonstrated that children who received 0.12% chlorhexidine gel in combination with tooth brushing had decreased colonization of pathogenic organisms such as Klebsiella pneumoniae and Pseudomonas aeruginosa. Infection is a major factor in patient outcomes, as evidenced by the statistically significant correlation between mortality and a positive sputum culture (*p* = 0.002). Crucially, culture positivity was significantly associated with mortality, particularly in the control group (*p* = 0.006), but this association decreased in the intervention group (*p* = 0.129), which may suggest that improved oral hygiene has a protective effect.

Moreover, de Camargo et al. [12], reported that the reduced microbial colonization observed was associated with lower VAP-related complications and a trend toward reduced mortality. These findings suggest that improved oral hygiene may have a protective effect by reducing infection burden rather than changing systemic inflammation, which could account for the weaker correlation between culture positivity and mortality in the current study’s intervention group.

The higher prevalence of multidrug-resistant (MDR) organisms in the control group including higher proportions of Pseudomonas aeruginosa, Klebsiella pneumoniae, and Acinetobacter baumannii is a clinically significant finding that warrants particular attention in the context of antibiotic stewardship. MDR pathogens are associated with treatment failure, prolonged hospitalization, and increased mortality, and their emergence is closely linked to antibiotic selective pressure and inadequate infection control measures. These findings suggest that enhanced mechanical oral hygiene through toothbrushing may reduce not only the overall burden of microbial colonization but also the selective proliferation of resistant organisms. This has direct implications for antibiotic prescribing practices and infection control protocols in PICU settings, where MDR pathogens represent a major and growing challenge. Prospective studies incorporating formal antimicrobial resistance profiling and antibiotic consumption data are needed to confirm this relationship.

Neither age nor gender significantly influenced mortality or infection risk in this cohort. Although a trend toward higher mortality among females in the intervention group was observed (*p* = 0.06), this did not reach statistical significance. To further explore this finding, univariate and multivariate logistic regression analyses were performed for mortality predictors, including sex, age, group allocation, culture positivity, inotropic support requirement, and new pulmonary infiltrates. Sex did not emerge as an independent predictor of mortality after adjusting for culture positivity and inotropic support, suggesting that the observed gender-based trend is likely confounded by differences in underlying illness severity rather than reflecting a true biological effect. This is consistent with Iosifidis et al. [13], who concluded that age and gender were not independently associated with increased VAP risk or mortality, whereas duration of mechanical ventilation and comorbidities were the key predictive factors.

### Cost-effectiveness analysis

The cost-effectiveness was calculated based only on the cost of oral care materials. This analysis reflects direct material costs only and does not include PICU stay or staffing costs. In the control group, routine oral care materials cost about 11,750 EGP (≈ 235 USD) for 5 days, while in the intervention group, tooth brushing with chlorhexidine cost only 70 EGP (≈ 1.4 USD) for the same period. This highlights the minimal cost of the intervention while potentially supporting better clinical outcomes.

### Limitations of the study

To our knowledge, the majority of data currently available on oral hygiene for the prevention of VAP is from adult populations, and this remains a relatively underexplored area in pediatric intensive care. However, this study has several limitations that need to be considered.

First, the diagnostic criteria applied in this study are based on the traditional CDC/NHSN clinical definition of VAP, which predates the current Ventilator-Associated Events (VAE) surveillance framework. The newer framework classifies events on a spectrum (VAC → IVAC → Possible/Probable VAP) and may yield different event rates. This discrepancy should be considered when comparing our findings to studies using the current VAE taxonomy [14].

Second, chest radiographs were interpreted by the treating PICU team rather than an independent radiologist, which may have introduced subjective assessment bias in the identification of new pulmonary infiltrates.

Third, a validated illness severity scoring system (such as PRISM III or PELOD-2) was not incorporated into the study design. This limits the ability to fully characterize baseline illness severity and to adjust for it in outcome comparisons. Future studies should include validated severity scoring as a routine baseline variable.

Fourth, although nursing staff received structured training and adherence was monitored informally throughout the study, formal quantitative compliance data such as the proportion of sessions completed per patient were not prospectively recorded. This represents a potential source of performance bias and limits our ability to quantify the relationship between protocol adherence and clinical outcomes.

Fifth, some patients died before completing the oral care protocol and sputum culture collection, which may have affected data completeness.

Finally, the study was conducted in a single center with a relatively small sample size, which may limit generalizability. Several outcomes did not reach statistical significance despite trends favoring the intervention. These results need to be confirmed by larger multicenter studies.

## Conclusion

This study revealed that implementing an enhanced oral hygiene protocol involving tooth brushing combined with 0.12% chlorhexidine mouthwash in mechanically ventilated pediatric patients may contribute to a reduction in the incidence of ventilator-associated pneumonia, as evidenced by lower rates of positive sputum cultures, a decreased need for inotropic support, and fewer new pulmonary infiltrates than those associated with routine chlorhexidine swabbing alone. Although differences in mortality and other clinical outcomes between the intervention and control groups did not reach statistical significance, findings suggest a possible clinical benefit that warrants confirmation in larger multicenter trials. According to these results, tooth brushing is an easy, affordable, and low-risk addition to routine oral care in the PICU. Larger multicenter studies are needed to confirm these findings and further evaluate the impact of enhanced oral hygiene on long-term clinical outcomes.

## Supplementary Information

Supplementary Material 1.

Supplementary Material 2.

Supplementary Material 3.

## Data Availability

The datasets generated and/or analysed during the current study are available from the corresponding author on reasonable request.

## Abbreviations

VAP: Ventilator-associated pneumonia
CDC: Centers for Disease Control and Prevention
NHSN: National Healthcare Safety Network
CRP: C-reactive protein
TLC: Total leukocyte count
VBG: Venous blood gases
BAL: Broncho-alveolar lavage
CBC: Complete blood count
MV: Mechanical ventilation
SD: Standard deviation
IQR: Interquartile range
