# Supplementary material for: Tooth brushing versus routine oral care impact on ventilator-associated pneumonia in PICU: a clinical trial
Source: BMC Pediatr. 2026 Jul 24;26:683. doi: 10.1186/s12887-026-07239-x (PMC13401315; doi:10.1186/s12887-026-07239-x)
Supplement: Supplementary file 3 — Supplementary Material 3. [file 12887_2026_7239_MOESM3_ESM.docx]

**Appendix 1 Ethical Approval given by the research ethics committee (REC), Faculty of Medicine Ain Shams University**

**
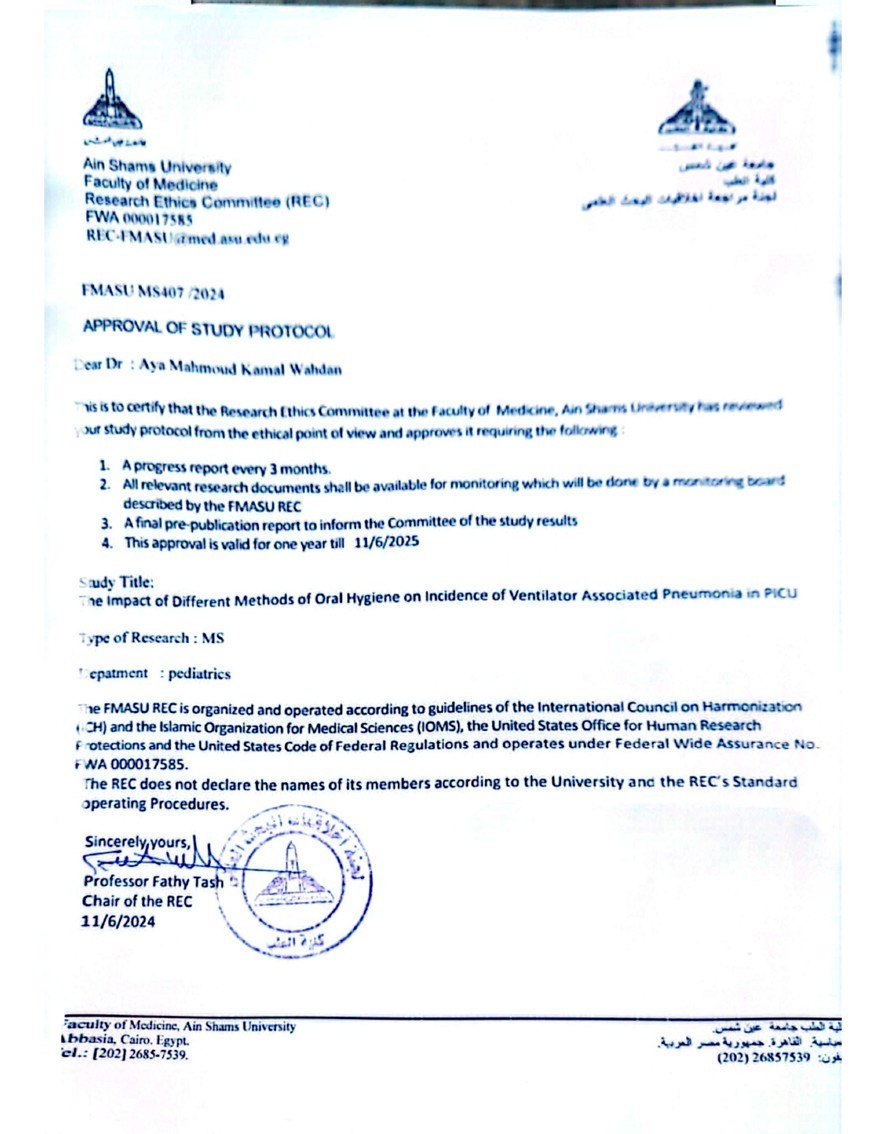
**

**Appendix 2 Guardian-informed consent**

Ain Shams University Faculty of Medicine

Ethical Committee of Scientific research

Informed consent form for parents or guardians of patients who are invited to participate in the research

Research title: Tooth brushing versus routine oral care impact on ventilator-associated pneumonia in PICU: A clinical Trial

Introduction and aim of the work:

Ventilator-associated pneumonia (VAP) is defined by the Center for Disease Control and Prevention (CDC) and National Healthcare Safety Network (NHSN) as new and persistent radiographic infiltrates and worsening gas exchange in children who are ventilated for at least 48 hours and who exhibit at least 3 of the following criteria: temperature instability with no other recognized cause, leukopenia, change in the characteristic of respiratory secretions, respiratory distress and bradycardia or tachycardia *(Semenkovich et al., 2019).*

VAP is the second most common hospital-acquired infection among PICU patients. It accounts for 7% to 32% of healthcare-associated infections and 10% of all pediatric device-related infections reported to NHSN. *(Bhattacharya et al., 2023).*

The aim of the work is to determine incidence of VAP in patients who will be admitted to PICU with routine oral hygiene in comparison to those using brushing and chlorhexidine. To detect the impact of different methods of oral hygiene on mechanically ventilated patients regarding duration of mechanical ventilation, length of PICU stay and mortality rate.

Place of work:

Ain-Shams University Children hospital, Pediatric Intensive Care Units (PICUs).

Number and Selection of participants:

- 118 pediatric patients.

Plan of the work:

The following assessment will be done to each patient.

1. History:
   - Demographic data: (name, age, sex).
   - Medical diagnosis on admission.
   - Comorbidities.
   - Need for inotropic support.
   - Day of worsening of respiratory symptoms after ventilation.
   - Fate

Risk factors:

- - Days in (PICU) until intubation*.*
  - Days on mechanical ventilation (MV) until infection*.*
  - Potential risk factors: inhaled bronchodilators, proton pump inhibitors, neuro-muscular blockers (doses & duration).
  - Route of mechanical ventilation: Nasotracheal, endotracheal, tracheostomy*.*
  - Method of nutrition: Nasogastric tube, parenteral, gastrostomy.
  - The antibiotic used (name, including duration, need to switch)*.*

1- General examination*:* Routine monitoring of Temperature, Heart Rate, Respiratory Rate, Blood Pressure.

Physical examination: Chest auscultation.

Then patients will be divided into 2 groups (59 patients in each group).

Benefits expected from the study:

To determine incidence of VAP in patients who will be admitted to PICU with routine oral hygiene in comparison to those using brushing and chlorhexidine. To detect the impact of different methods of oral hygiene on mechanically ventilated patients regarding duration of mechanical ventilation, length of PICU stay and mortality rate.

*Conducting the consent:*

The consent will be conducted to the legal guardian or the patient by the investigator, Dr. Ahmed Rezk assistant professor in the Pediatrics Department, Ain Shams University Hospital. Literate individuals will be left to read the consent followed by its explanation by the mentioned investigator, while illiterate individuals will have the consent read and explained to them as well.

Risks and complications:

- No risks

Reimbursements in cases of risks and complications:

Should your patient get physically injured as a result of research- related procedures, Doctor Ahmed Rezk will provide first-aid medical treatment.

Alternatives to participating:

In case of refusing to participate in this research, your patient will be followed up and will receive his treatment as planned.

Confidentiality:

You will deal with complete confidentiality, and no one has right to read your patient medical information except the main researcher. After the research is complete, you will be informed regarding your patient `s research results and also further information regarding your patient `s health status.

Right to refuse or withdraw:

Any participant doesn`t have to take part in this research if he/she or want. They may also stop participating at any time. If you have read this form and have decided to let your patient participate in this study, please understand that your patient’s participation is voluntary and you have the right to withdraw your consent or discontinue participation at any time without penalty or loss of benefits to which your patient is otherwise entitled. Your decision whether to participate in this study will not affect your patient’s medical care. Individual privacy will be maintained in all published and written data resulting from the study.

Contact Information:

Questions, Concerns, or Complaints: If you have any questions, concerns or complaints about this research study, its procedures, risks and benefits, or alternative courses of treatment, you should ask the investigator, Dr. Ahmed Rezk, at mobile number: 01113311183. If you have any problems or concerns about the study, you can also call Dr. Nehad Bakry, and her contact number is 01090199302.

You do not have to sign this consent form. But if you do not, your patient will not be able to participate in this research study.

Certificate of consent:

I have read the foregoing information, or it has been read to me. I have had the opportunity to ask questions about it and any questions that I ask to have been answered to my satisfaction. I voluntarily consent to participate in this research and understand that I have the right to withdraw from the research at any time without in any way affecting my patient`s medical care.

- Name of participant:
- Signature of legal guardian:
- Or participant:
- Identity number or fingerprint: ………………………….
- Date:

I have accurately read or witnessed the accurate reading of the consent to the potential participant. The individual has had the opportunity to ask questions I confirm that the individual has given consent freely.

- Name of researcher: Ahmed Rezk
- Signature of researcher:
- Date:

This proposal has been reviewed and approved by Ethical Committee of Scientific Research, which is a committee whose task is to make sure that research participants are protected from harm.

If you wish to find out more about Ethical Committee of Scientific research.

Contact: Name: Address:

Telephone number:
