# Supplementary material for: Tooth brushing versus routine oral care impact on ventilator-associated pneumonia in PICU: a clinical trial
Source: BMC Pediatr. 2026 Jul 24;26:683. doi: 10.1186/s12887-026-07239-x (PMC13401315; doi:10.1186/s12887-026-07239-x)
Supplement: Supplementary file 1 — Supplementary Material 1. [file 12887_2026_7239_MOESM1_ESM.docx]

Dear Editor,

We would like to thank you for the opportunity to revise and resubmit our manuscript entitled:

“Tooth brushing versus routine oral care impact on ventilator-associated pneumonia in PICU: A clinical trial”

We have carefully addressed all the editorial comments and revised the manuscript accordingly. All changes have been clearly highlighted, and a detailed point-by-point response is provided.

The following key modifications have been made:

* The trial registration details were verified and corrected to match the ClinicalTrials.gov registry, and the statement “Retrospectively registered” has been added.

* The manuscript has been revised to fully comply with CONSORT 2010 guidelines, and a corresponding statement has been included in the Methods section.

* All required sections under “Declarations” have been completed and structured according to journal requirements.

* A data availability statement has been added.

* Minor revisions were performed to improve clarity, language, and formatting throughout the manuscript.

We believe that the revised manuscript has been significantly improved and now meets the journal’s requirements. We hope it will be considered suitable for publication in BMC Pediatrics.

Thank you for your time and consideration.

Kind regards,

Dr. Nehad Bakry

Consultant Pediatrician

Ain Shams University

Email: [nehad.bakry@med.asu.edu.eg](mailto:nehad.bakry@med.asu.edu.eg)
